# The association between the reduction of body weight and new-onset type 2 diabetes remission in middle-aged Japanese men: Population-based Panasonic cohort study 8

**DOI:** 10.3389/fendo.2022.1019390

**Published:** 2023-01-16

**Authors:** Takaaki Matsui, Hiroshi Okada, Masahide Hamaguchi, Kazushiro Kurogi, Hiroaki Murata, Masato Ito, Michiaki Fukui

**Affiliations:** ^1^ Department of Endocrinology and Metabolism, Kyoto Prefectural University of Medicine, Graduate School of Medical Science, Kyoto, Japan; ^2^ Department of Diabetes and Endocrinology, Matsushita Memorial Hospital, Moriguchi, Japan; ^3^ Department of Health Care Center, Panasonic Health Insurance Organization, Moriguchi, Japan; ^4^ Department of Orthopaedic Surgery, Matsushita Memorial Hospital, Moriguchi, Japan

**Keywords:** type 2 diabetes, body weight loss, diabetes remission, medical health checkup, obesity

## Abstract

**Aim:**

This study aimed to investigate the association between change in body weight (BW) and type 2 diabetes remission in Japanese men with new-onset type 2 diabetes.

**Methods:**

This study enrolled 1,903 patients with new-onset type 2 diabetes between 2008 and 2013 from a medical health checkup program conducted by the Panasonic Corporation, Osaka, Japan. The baseline was defined as the year of new-onset diabetes. We assessed the type 2 diabetes remission five years after baseline and the association between the change in BW and type 2 diabetes remission using logistic regression analyses. To evaluate the predictive performance of the change in BW, we employed the receiver operating characteristic curves and the area under the receiver operating characteristic (ROC) curve (AUC).

**Results:**

The BW loss was associated with type 2 diabetes remission in the participants with a BMI ≥25 kg/m^2^ but not in the participants with a BMI <25 kg/m^2^. The odds ratios were 1.96 (95% CI: 1.19–3.29) and 3.72 (95% CI: 2.14–6.59) in the participants with a loss of 5–9.9% and loss of ≥10% for five years, respectively, in the participants with a BMI ≥25 kg/m^2^ (reference; stable group [0.9% gain to 0.9% loss]). The AUC and cut-off values for the rate of change in BW for type 2 diabetes remission were 0.59 and 5.0%.

**Discussion:**

Body weight loss of ≥5% effectively achieved diabetes remission in Japanese men with a BMI ≥25 kg/m^2^ and new-onset type 2 diabetes.

## Introduction

There are several well-known risk factors for the onset of diabetes in the Japanese population, such as hyperlipidemia, hypertension, aging, weight gain, smoking history, impaired glucose tolerance (IGT), and family history (1–8). One of the goals in clinical care is the prevention of diabetes by focusing on these risk factors. However, in recent decades, the number of patients with diabetes and medical costs of diabetes have been increasing worldwide. Therefore, diabetes remission is as important as diabetes prevention.

Several Japanese studies have reported the association between body weight loss and diabetes prevention in patients with IGT. Kosaka et al. (9) conducted an intervention trial on whether body weight reduction by diet and exercise could prevent progression to diabetes among male patients with IGT in an outpatient clinic. Their study showed that the reduction in risk of diabetes was 67.4% lower in the intervention group than in the control group, and body weight loss was higher in the intervention group than in the control group (loss of 2.18 kg versus 0.39 kg) for 4 years. Kawahara et al. (10) conducted an intervention trial on whether a short-term hospital program of diabetes education could prevent progression to diabetes in patients with IGT. They reported that the incidence of diabetes was 42% lower in the intervention group than in the control group for 3 years. They also observed that body weight loss was higher in the intervention group than in the control group (loss of 2.1 kg versus gain of 0.4 kg). Furthermore, Saito et al. (11) conducted an intervention trial involving patients with IGT in an outpatient clinic. They reported that the risk of incident diabetes was 59% lower in the intervention group than that in the control group and that body weight loss was higher in the intervention group than in the control group (loss of 2.5 kg versus 1.1 kg). Thus, the association between body weight loss and prevention of type 2 diabetes in patients with IGT might be obvious. However, to our knowledge, there are no studies that assessed the association between change in body weight and diabetes remission in Japanese patients with type 2 diabetes. The findings of such studies may help in setting strategies to achieve type 2 diabetes remission. This is the first study to investigate the association between change in body weight and type 2 diabetes remission in Japanese men with new-onset type 2 diabetes.

## Materials and methods

### Study design and data collection

This retrospective cohort study included participants of a physical examination program at Panasonic Corporation, Osaka, Japan. This study was named Panasonic cohort study and used 2008–2018 data from Panasonic Corporation’s database. All the participants partook in the physical examination program yearly from 2008 to 2018. The baseline was defined as the year of new-onset diabetes. The participants’ baseline characteristics were evaluated using a self-administered questionnaire. The participants were classified into current smokers, past smokers, and non-smokers based on smoking habits. The participants who regularly practiced any sport twice a week for more than one year were classified as regular exercisers.

The study was approved by the local ethics committee of Panasonic Health Insurance Organization (Approval number: 2021-001) and was conducted in accordance with the principles of the Declaration of Helsinki.

### Change in body weight

Body weight and height of all participants were recorded using an automatic machine yearly. We collected body weight five years after baseline to evaluate the change in this variable. Change in body weight was calculated as follows: body weight five years after baseline – body weight at baseline. The rate of change in body weight (%) was calculated as follows: (body weight five years after baseline – body weight at baseline) × 100/body weight at baseline.

### Definitions of onset and remission of type 2 diabetes

Participants with a fasting plasma glucose concentration ≥126 mg/dL and/or who were on antihyperglycemic medication were considered as having type 2 diabetes. Participants with a fasting plasma glucose concentration <126 mg/dL and who were not taking antihyperglycemic medication were considered as having type 2 diabetes remission. We calculated the incidence of new-onset type 2 diabetes between 2008 and 2013 among the participants who did not have diabetes in 2008 and type 2 diabetes remission five years after baseline to evaluate the association between the change in body weight and type 2 diabetes remission in the participants with new-onset type 2 diabetes.

### Exclusion criteria

Overall, 84,997 men partook in the physical examination program in 2008. We excluded the participants with diabetes in 2008 (n = 4943). Of 80,054 men without diabetes in 2008, 3,264 men developed type 2 diabetes between 2008 and 2013. Of 3,264 men with new-onset type 2 diabetes, we excluded 1361 men who did not partake in the physical examination program five years after baseline. The final analysis involved the data of 1,903 men with new-onset diabetes between 2008 and 2013.

### Statistical analyses

The differences in the general characteristics at baseline (the year of new-onset diabetes) according to the type 2 diabetes remission were evaluated using Student’s t-test and chi-square test, as appropriate. The association between the change in body weight and type 2 diabetes remission was assessed by logistic regression analyses. The multivariate analysis was adjusted for the factors related to type 2 diabetes, such as body mass index (BMI), age, systolic blood pressure (SBP), serum high-density lipoprotein (HDL) cholesterol concentrations, serum low-density lipoprotein (LDL) cholesterol concentrations, serum triglycerides concentrations, serum fasting plasma glucose concentrations, serum uric acid concentrations, smoking status, physical exercise habits, and change in body weight. The categorical values of the change in body weight and the rate of change in body weight were added into multivariate models to assess the association between categorical values and type 2 diabetes remission. The five groups according to change in body weight (gain [≥ 1 kg gain], stable [0.9 kg gain to 0.9 kg loss], loss of 1–4.9 kg, loss of 5–9.9 kg, and loss of ≥10 kg) and the rate of change in body weight (gain [≥ 1% gain], stable [0.9% gain to 0.9% loss], loss of 1–4.9%, loss of 5–9.9%, and loss of ≥10%) were added to the multivariate analyses of data of all the participants and participants with obesity (BMI ≥25 kg/m^2^) at baseline, respectively. The four groups according to change in body weight (gain [≥ 1 kg gain], stable [0.9 kg gain to 0.9 kg loss], loss of 1–4.9 kg, and loss of ≥5 kg) and the rate of change in body weight (gain [≥ 1% gain], stable [0.9% gain to 0.9% loss], loss of 1–4.9%, and loss of ≥5%) were added to the multivariate analyses of data of non-obese participants (BMI <25 kg/m^2^) at baseline, respectively. A receiver operating characteristic curve analysis was performed for change in body weight to assess the ability to identify patients with type 2 diabetes remission. We used JMP software (SAS Institute, NC, USA) to performe all statistical analyses. Continuous variables are expressed as mean ± standard deviation or absolute numbers. P values <0.05 were considered statistically significant. The associations are presented as hazard ratios with 95% confidence intervals (CIs).

## Results

**Figure 1:**
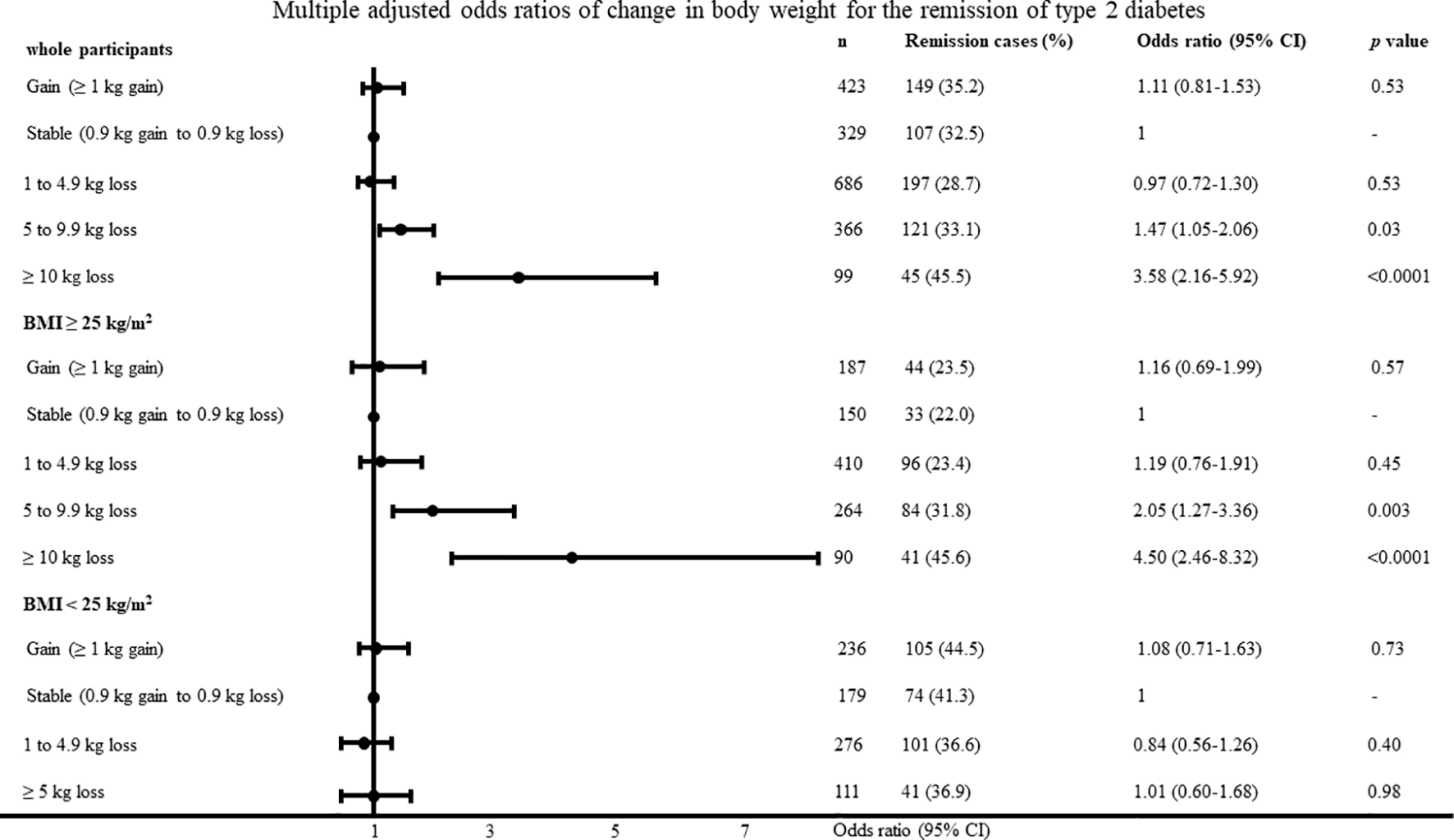
Multiple adjusted odds ratios of change in body weight for the remission of type 2 diabetes adjusted for age, body mass index, systolic blood pressure, low-density lipoprotein cholesterol, high-density lipoprotein cholesterol, triglyceride, fasting plasma glucose, uric acid, smoking habit and physical activity.

**Figure 2:**
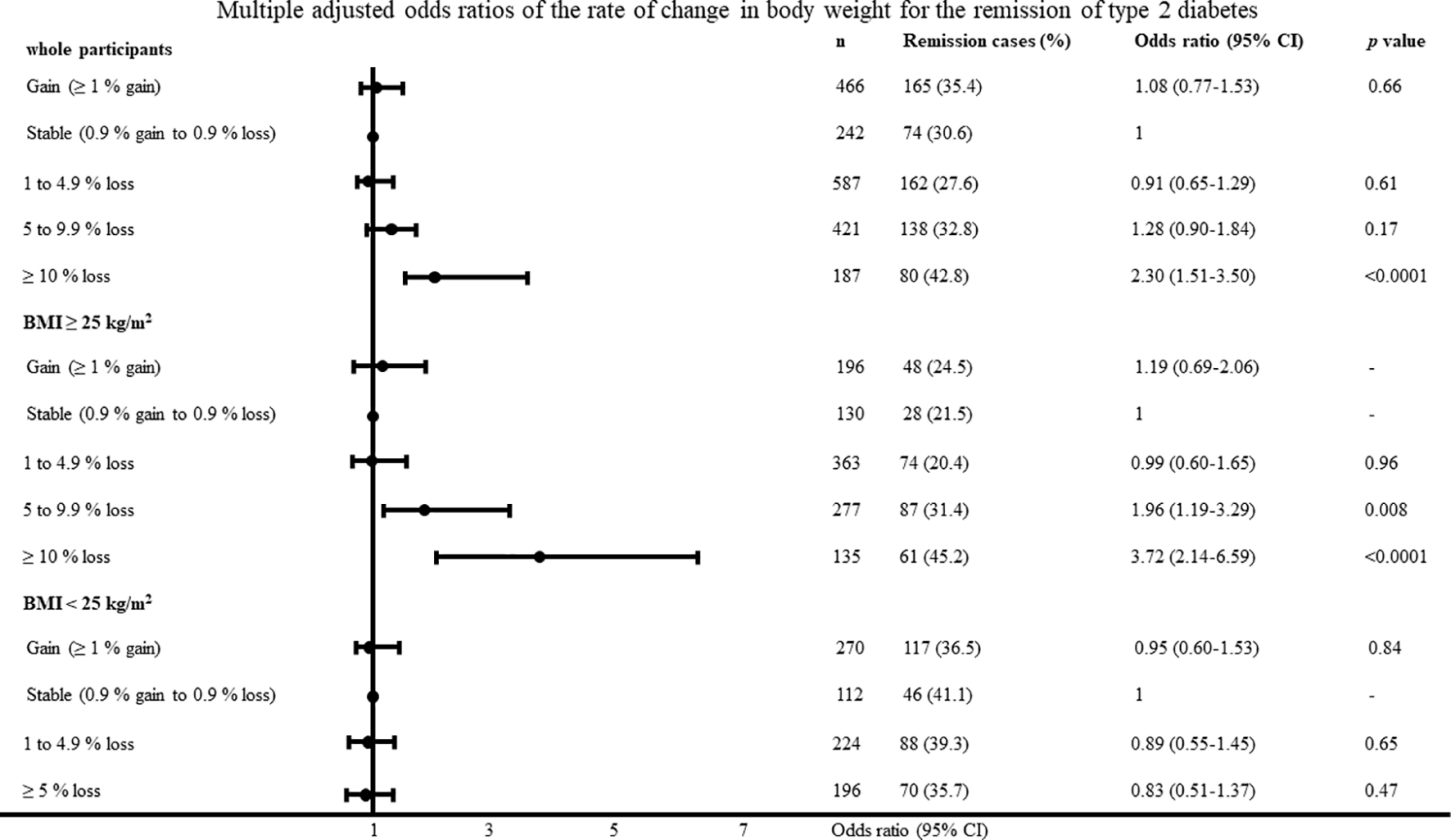
Multiple adjusted odds ratios of change in body weight for the remission of type 2 diabetes adjusted for age, body mass index, systolic blood pressure, low-density lipoprotein cholesterol, high-density lipoprotein cholesterol, triglyceride, fasting plasma glucose, uric acid, smoking habit and physical activity.

The baseline characteristics of all the participants with new-onset type 2 diabetes are shown in **Table 1**. In total, 619 participants (32.5%) had type 2 diabetes remission five years after baseline. In the participants with a BMI ≥25 kg/m^2^ and BMI < 25 kg/m^2^, 298 (27.1%) and 321 (40.0%) participants had type 2 diabetes remission five years after baseline. The average change in body weight was -2.3 ± 4.9 kg, -3.3 ± 5.5 kg, and -0.9 ± 3.8 kg in the overall participants, participants with BMI ≥25 kg/m^2^, and participants with BMI <25 kg/m^2^. The proportions of participants with type 2 diabetes remission in the overall study population are shown in **Figures 1** and **2**. The proportion of type 2 diabetes remission varied with the degree of change in body weight loss in the overall participants and the participants with a BMI ≥25 kg/m^2^.

**Table 1:** Characteristics of participants at baseline according to type 2 diabetes remission.

	All	Remission of diabetes (-)	Remission of diabetes (+)	P value
N	1,903	1,284	619	–
Age (y)	48.3 (5.3)	48.3 (5.0)	48.1 (5.8)	0.35
Body mass index (kg/m^2^)	26.3 (4.2)	26.8 (4.2)	25.1 (4.0)	<0.0001
Systolic blood pressure (mmHg)	128.0 (14.5)	128.2 (14.7)	127.5 (14.2)	0.29
Diastolic blood pressure (mmHg)	81.5 (10.4)	82.0 (10.3)	80.5 (10.5)	0.004
LDL cholesterol (mg/dL)	134.0 (33.8)	137.0 (33.0)	127.7 (34.5)	<0.0001
HDL cholesterol (mg/dL)	51.9 (12.8)	50.9 (12.2)	54.1 (13.9)	<0.0001
Triglycerides (mg/dL)	184.9 (154.6)	188.7 (144.5)	177.0 (173.4)	0.12
Glucose (mg/dl)	136.6 (25.4)	138.4 (28.4)	132.9 (17.1)	<0.0001
Uric acid (mg/dL)	6.1 (1.5)	6.2 (1.4)	6.1 (1.7)	0.41
Smoking (none/past/current)	714/412/777	468/283/533	246/129/244	0.38
Physical exercise (+/-)	342/1561	232/1,052	110/509	0.87

Data are presented as mean (standard deviation) or absolute number.

LDL, low-density lipoprotein; HDL, high-density lipoprotein.

The unadjusted and adjusted odds ratios in the multivariate models for type 2 diabetes remission are shown in **Table 2**. For every 1 kg reduction in body weight for 5 years, the odds ratio of type 2 diabetes remission increased by 6% in the overall participants. The adjusted odds ratios in the multivariate models for type 2 diabetes remission according to BMI category are shown in **Table 3**. For every 1 kg reduction in body weight for 5 years, the odds ratio of type 2 diabetes remission increased by 9% in the participants with a BMI ≥25 kg/m^2^. Conversely, the degree of reduction in body weight was not associated with type 2 diabetes remission in the participants with a BMI <25 kg/m^2^. The BMI, LDL cholesterol, fasting plasma glucose, and smoking habit were associated with type 2 diabetes remission in the participants with a BMI <25 kg/m^2^.

**Table 2:** Unadjusted and adjusted odds ratios for type 2 diabetes remission.

	Crude		Multiple	
	Odds ratio (95% CI)	P value	Odds ratio (95% CI)	P value
Age (per 10years)	0.92 (0.76-1.10)	0.35	0.72 (0.59-0.88)	0.001
Body mass index (per 1kg/m^2^)	0.90 (0.88-0.92)	<0.0001	0.89 (0.86-0.92)	<0.0001
Systolic blood pressure (per 10mmHg)	0.96 (0.90-1.03)	0.29	1.03 (0.96-1.11)	0.41
LDL cholesterol (per 10mg/dl)	0.92 (0.89-0.95)	<0.0001	0.93 (0.90-0.96)	<0.0001
HDL cholesterol (per 10mg/dl)	1.20 (1.12-1.30)	<0.0001	1.08 (0.99-1.18)	0.08
Triglycerides (per 10mg/dl)	0.995 (0.99-1.001)	0.11	0.999 (0.99-1.007)	0.87
Glucose (per 10mg/dl)	0.90 (0.85-0.94)	<0.0001	0.91 (0.86-0.96)	0.0001
Uric acid (per 1mg/dl)	0.97 (0.91-1.04)	0.41	1.02 (0.95-1.09)	0.59
Change in body weight (per 1kg loss)	1.02 (0.999-1.04)	0.06	1.06 (1.04-1.08)	<0.0001
Smoking (past) (ref: none)	0.87 (0.67-1.12)	0.28	0.82 (0.62-1.07)	0.14
Smoking (current) (ref: none)	0.87 (0.70-1.08)	0.21	0.83 (0.66-1.05)	0.12
Physical exercise (yes) (ref: no)	0.98 (0.76-1.23)	0.87	0.91 (0.70-1.18)	0.48

LDL, low-density lipoprotein; HDL, high-density lipoprotein.

**Table 3:** Adjusted odds ratios for type 2 diabetes remission according to BMI category.

	BMI ≥ 25 kg/m^2^ (n = 1,101)		BMI < 25 kg/m^2^ (n = 802)	
	Odds ratio (95% CI)	P value	Odds ratio (95% CI)	P value
Age (per 10years)	0.70 (0.53-0.92)	0.01	0.77 (0.57-1.03)	0.08
Body mass index (per 1kg/m^2^)	0.89 (0.85-0.94)	<0.0001	0.80 (0.73-0.88)	<0.0001
Systolic blood pressure (per 10mmHg)	0.99 (0.89-1.10)	0.84	1.08 (0.98-1.20)	0.13
LDL cholesterol (per 10mg/dl)	0.93 (0.89-0.97)	0.002	0.93 (0.89-0.98)	0.002
HDL cholesterol (per 10mg/dl)	1.18 (1.02-1.36)	0.02	1.002 (0.89-1.12)	0.98
Triglycerides (per 10mg/dl)	0.998 (0.99-1.008)	0.70	1.002 (0.99-1.01)	0.65
Glucose (per 10mg/dl)	0.90 (0.83-0.97)	0.005	0.91 (0.84-0.97)	0.004
Uric acid (per 1mg/dl)	1.04 (0.94-1.14)	0.47	1.03 (0.93-1.15)	0.57
Change in body weight (per 1kg loss)	1.09 (1.06-1.12)	<0.0001	0.99 (0.95-1.03)	0.57
Smoking (past) (ref: none)	0.88 (0.60-1.27)	0.49	0.72 (0.48-1.08)	0.11
Smoking (current) (ref: none)	0.95 (0.69-1.32)	0.78	0.68 (0.49-0.97)	0.03
Physical exercise (yes) (ref: no)	0.91 (0.62-1.32)	0.63	0.87 (0.59-1.26)	0.46

LDL, low-density lipoprotein; HDL, high-density lipoprotein.

The multiple adjusted odds ratios of change in body weight and rate of change in body weight according to the categorical values for type 2 diabetes remission with the body weight stable group as reference are shown in **Figures 1** and **2**. In the participants with a BMI ≥25 kg/m^2^, the odds ratios were 2.05 (95% CI, 1.27–3.36; P = 0.003) and 4.50 (95% CI, 2.46–8.32; P <0.0001) in the participants with a loss of 5–9.9 kg and ≥10 kg, respectively. In the participants with a BMI <25 kg/m^2^, change in body weight, and rate of change in body weight was not associated with type 2 diabetes remission. The results were almost identified with the change in body weight when we evaluated odds ratios regarding the rate of change in body weight. When diabetes remission was defined as < 110mg/dl, the results was almost same as definition of diabetes remission < 126 mg/dl. In the participants with a BMI ≥25 kg/m^2^, the odds ratios were 7.96 (95% CI, 3.72–17.69; P < 0.0001) in the participants with a loss of ≥10 kg. When diabetes remission was defined as < 110mg/dl, change in body weight was not associated with type 2 diabetes remission in the participants with a BMI <25 kg/m^2^.

The area under the curve and cut-off values of the change in body weight and rate of change in body weight for type 2 diabetes remission were 0.58 and 3.9 kg loss, and 0.59 and 5.0% loss in the participants with a BMI ≥25 kg/m^2^, respectively.

## Discussion

This study assessed the association between the change in body weight and type 2 diabetes remission in participants with new-onset diabetes. The major findings of our study were as follows: (1) body weight loss was associated with new-onset type 2 diabetes remission in the participants with a BMI ≥25 kg/m^2^ (obese) but not in the participants with a BMI <25 kg/m^2^ (non-obese); and (2) in patients with a BMI ≥25 kg/m^2^ (obese), a body weight loss of ≥3.9 kg or ≥5.0% might be effective for new-onset type 2 diabetes remission. Our findings are largely consistent with the guidelines of the Japan Society for the Study of Obesity, which recommends a body weight loss of ≥3.0% in the participants with a 25 ≤ BMI < 35 kg/m^2^ and a body weight loss of ≥5.0% in the participants with a BMI ≥35 kg/m^2^.

Body weight is strongly associated with the development of diabetes in Western people and Japanese people (12). Several studies have reported the association between body weight loss and diabetes remission. The Diabetes Remission Clinical Trial (DiRECT) was conducted to assess effective body weight management for diabetes remission (13). DiRECT showed that almost half of the participants achieved diabetes remission at 12 months in the intervention group, whose average body weight loss was –10.0 ± 8.0 kg (13). However, because eligible participants in DiRECT had a BMI of ≥27 kg/m^2^, it is unclear whether those findings were similar to persons with a BMI <27 kg/m^2^. An observational study in Scotland reported that more than 5 kg of body weight loss was associated with diabetes remission (14). The strengths of their study were that it involved the Western population in general and used data from their register that included information of 99% of the patients with diabetes in their country. We should consider ethnic differences in diabetes etiology. It has been reported that the BMI cut-off value for incident type 2 diabetes was lower in the Asian population than in the Western population (15). The mean BMI was 35.1 ± 4.5 kg/m^2^ in DiRECT, and the median BMI was 30.9 (27.4-35.3) kg/m^2^ in the Scottish study; their findings are not applicable to Asian people. Moreover, it might be difficult for participants to achieve a body weight loss of 10 kg in 1 year in a clinical care setting. Therefore, our findings can address the target body weight loss in Japanese in a clinical care setting.

Interestingly, 619 (32.5%) participants achieved type 2 diabetes remission in this study. This rate of remission was higher than that in a Western population (the Scottish study). This might be due to the difference in BMI from the Western population and whether the participants had no new-onset or new-onset diabetes, which could be more prone to remission. A proportion of 42.1% of the participants in our study had a BMI <25.0 kg/m^2^ (non-obese). As expected, in these participants, body weight loss was not associated with new-onset type 2 diabetes remission. The findings in participants with a BMI <25.0 kg/m^2^ might be due to diminished insulin secretion, which is characteristic of patients with diabetes in Asian countries (16). In the participants with a BMI <25.0 kg/m^2^, lipid disorder, increased plasma glucose concentration, and smoking habits might be more important than the change in body weight in new-onset type 2 diabetes remission. A body weight loss in participants with a BMI <25.0 kg/m^2^ might be associated with the comorbidity including malignancy, resulting in no improvement of diabetes remission. We found ten patients who have malignancy both in participants with BMI <25 kg/m^2^ and with BMI ≥25 kg/m^2^. Our results were almost the same when the participants with malignancy were excluded.

It has been proposed that the development of diabetes is triggered by insulin resistance, which eventually leads to the exhaustion of pancreatic β cells (17). Obesity reportedly induces chronic inflammation (18) and insulin resistance (19, 20), which is partly attributed to the dysregulation of adipocytokines such as tumor necrosis factor-α, adiponectin, leptin, and plasminogen activator inhibitor-1 (21, 22). The increased visceral adipose tissue, causing the adipocytes to produce more tumor necrosis factor-α and less adiponectin is associated with weight gain. It has been reported that body weight loss beneficially affects adipocytokines (23). Hence, participants with a BMI ≥25 kg/m^2^ and new-onset diabetes needed to lose body weight in the early disease stage to achieve type 2 diabetes remission.

The strengths of our study include its long follow-up period, real-world nature, and consecutive enrolment. However, this study had several limitations. First, in general, the diagnosis is mainly judged by plasma glucose and HbA1c. The diabetes remission is mainly defined as HbA1c below the level of 6.5% and remaining at that level for at least 3 months without continuation of the usual antihyperglycemic medication (24). However, diagnosis and remission of diabetes were judged only by fasting plasma glucose level and use of antihyperglycemic medication, but not HbA1c. Second, change in body composition rather than body weight may be more important for remission (25). However, we have no data about body composition. Thirdly, education on diabetes could have affected the rate of diabetes remission; however, we had no such data. Lastly, our study population was made up of relatively young Japanese men. We have no data in elder participants because our data was derived from cohort of a physical examination program at Panasonic Corporation. Therefore, it is unclear whether our findings are generalizable to women, other ethnic groups and age groups.

In conclusion, our study found that a body weight loss of ≥3.9 kg or ≥5.0% effectively achieved diabetes remission in Japanese men with a BMI ≥25 kg/m^2^ and new-onset type 2 diabetes. Therefore, it is important to focus on early body weight loss in participants with a BMI ≥25 kg/m^2^ and new-onset diabetes in clinical settings to achieve diabetes remission.

## Data availability statement

The raw data supporting the conclusions of this article will be made available by the authors, without undue reservation.

## Author contributions

TM wrote this manuscript. KK, MH, and HM contributed to the discussion. HO analyzed the data. HO and MI collected the data and contributed to the design and discussion. HO and MF edited and reviewed the manuscript. All authors contributed to the article and approved the submitted version.
